# Supplementary material for: Identification of a sugarcane bacilliform virus promoter that is activated by drought stress in plants
Source: Commun Biol. 2024 Mar 26;7:368. doi: 10.1038/s42003-024-06075-y (PMC10965894; doi:10.1038/s42003-024-06075-y)
Supplement: Supplementary file 2 — Supplementary Information [file 42003_2024_6075_MOESM2_ESM.pdf]

Supplementary Table 1. List of primers used in this study for gene/fragment cloning and qPCR assay.

Targeted gene/fragment	Primer name	Primer sequence (5'-3')
Partial sequence of SCBV genome	SCBV-AF5603	GAAGAGYGGSTTTCATCAAGT
	SCBV-AR1002	CTCCGCTTCAGGTATTCCW
ScbZIP72	ScbZIP72-F	ATGGACGAGCTGCTCCGGAGCATC
	ScbZIP72-R	TCAAGATAATATACAGGGGTC
ABER1	AT-ABER1-F	ATGGATGGTAGTATGAATTTGG
	AT-ABER1-R	TCACCAAGGTCCCGACTCTGTCCT
Gus	qGUS-F	AGCGTTGAACTGCGTGAT
	qGUS-R	TTGCCAGAGGTGCGGATT
Actin	qactin-F	TGTTCCCATCAGAACCGTGA
	qactin-R	CACCTGTCTTTGGGTCAACAA
ScbZIP72	qScbZIP72-F	CAGGCTTATACAATGGAGTTAG
	qScbZIP72-R	AACAGTTACCTCGTTCTTCT
GAPDH	qGAPDH-F	CACGGCCACTGGA AGCA
	qGAPDH-R	TCCTCAGGGTTCCTGATGCC
AtRD29A promoter	PAtRD29A-F	AGATCATACCTATTAGAACGATT
	PAtRD29A-F	TCCAATAGAAGTAATCAAACCCT
ScRD29A promoter	PScRD29A-F	CAGTGAAACAAACAGGGCCACA
	PScRD29A-R	CGCCGTCGACGTCGACGCGAAC

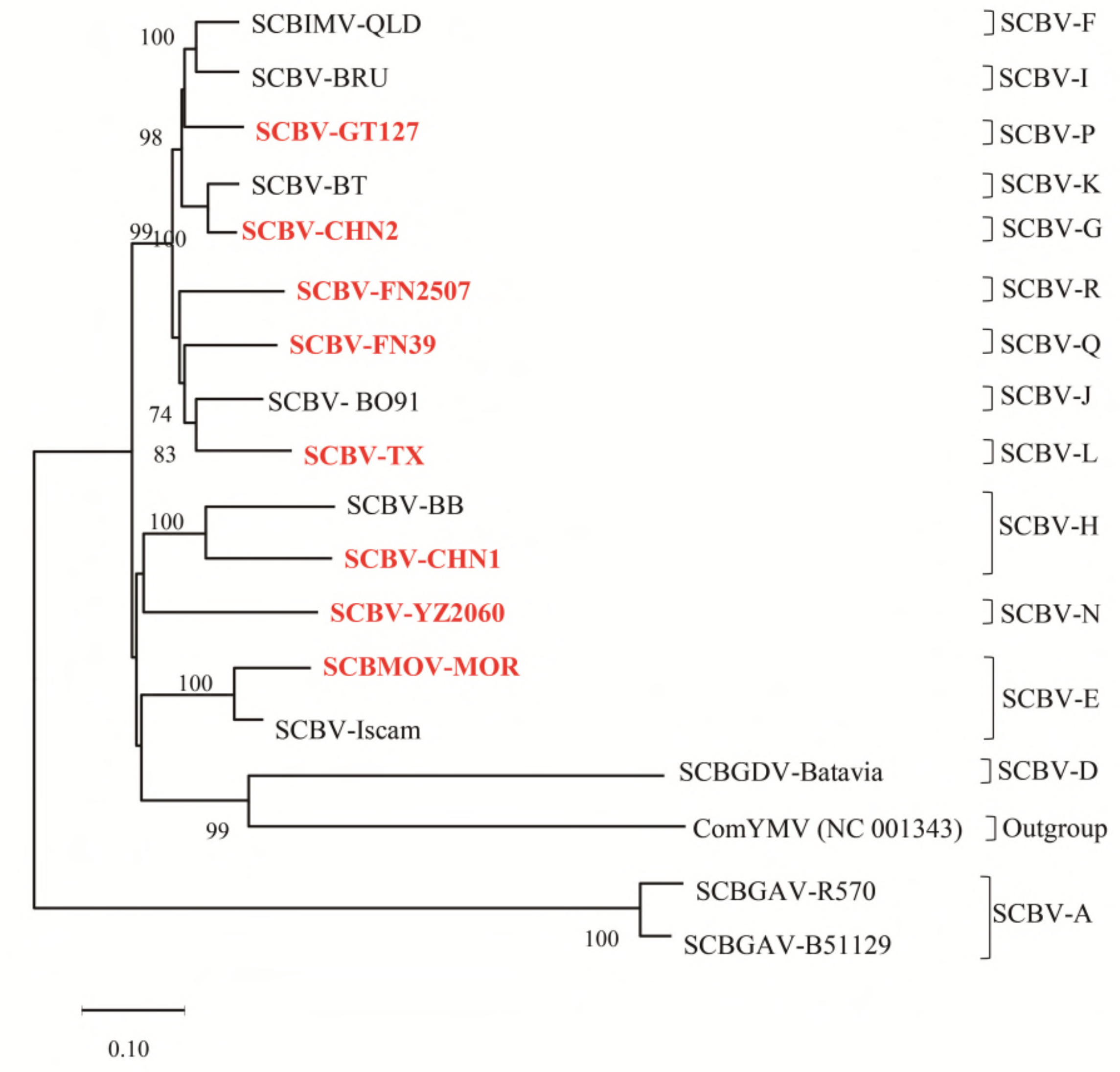

Supplementary Figure 1. Phylogenetic tree constructed with the sequence of a 3.0 kb partial genomic fragment of 17 SCBV isolates using the neighbor-joining (NJ) method. This fragment contained a partial gene coding for the RT/RNase H and the entire promoter region. Isolates in red were sequenced in this study and the other isolates were retrieved from GenBank: SCBIMV-QLD (NC_003031), SCBV-BRU (JN377537), SCBV-BT (JN377536), SCBV-BO91 (JN377533), SCBV-BB (JN377535), SCBV-Iscam (JN377534), SCBGDV-Batavia (FJ439817), SCBGAV-R570 (FJ824813), and SCBGAV-B51129 (FJ824814). Isolate ComYMV (NC 001343) was used as outgroup. Bootstrap values (1,000 replicates) are indicated at tree nodes and values < 60% were collapsed. Scale bar is in number of substitutions per nucleotide. Classification of SCBV genotypes was performed according to Janiga et al. (2023).

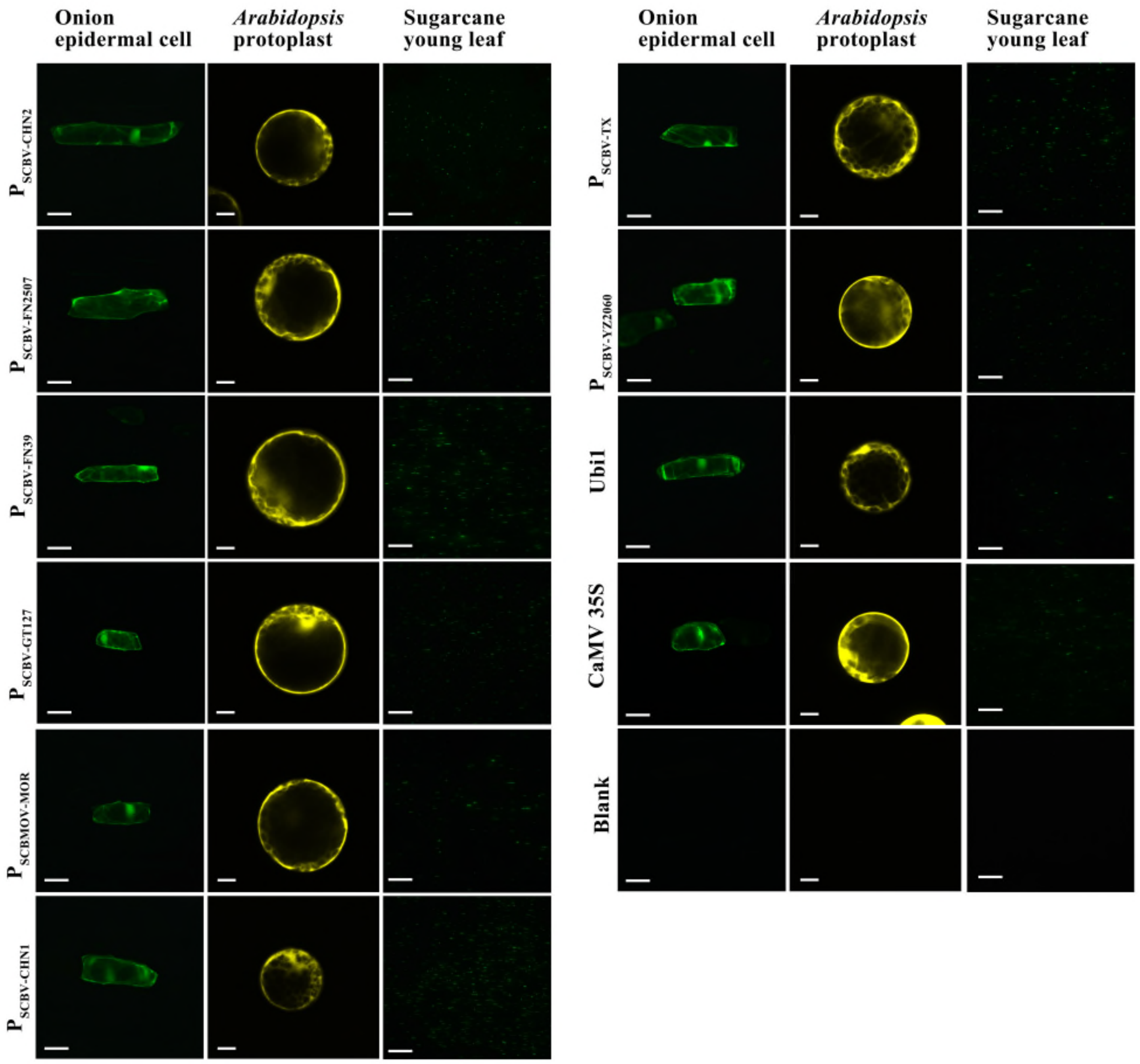

Supplementary Figure 2. Transient expression of *EYFP* reporter gene driven by eight SCBV promoters in onion epidermal cells, *Arabidopsis* mesophyll protoplasts, and young sugarcane leaf tissue. Scale bar represents 100 µm for onion cells, 25 µm for *Arabidopsis* protoplasts, and 250 µm for sugarcane leaf tissue. Ubi1 and CaMV 35S were used as positive controls.

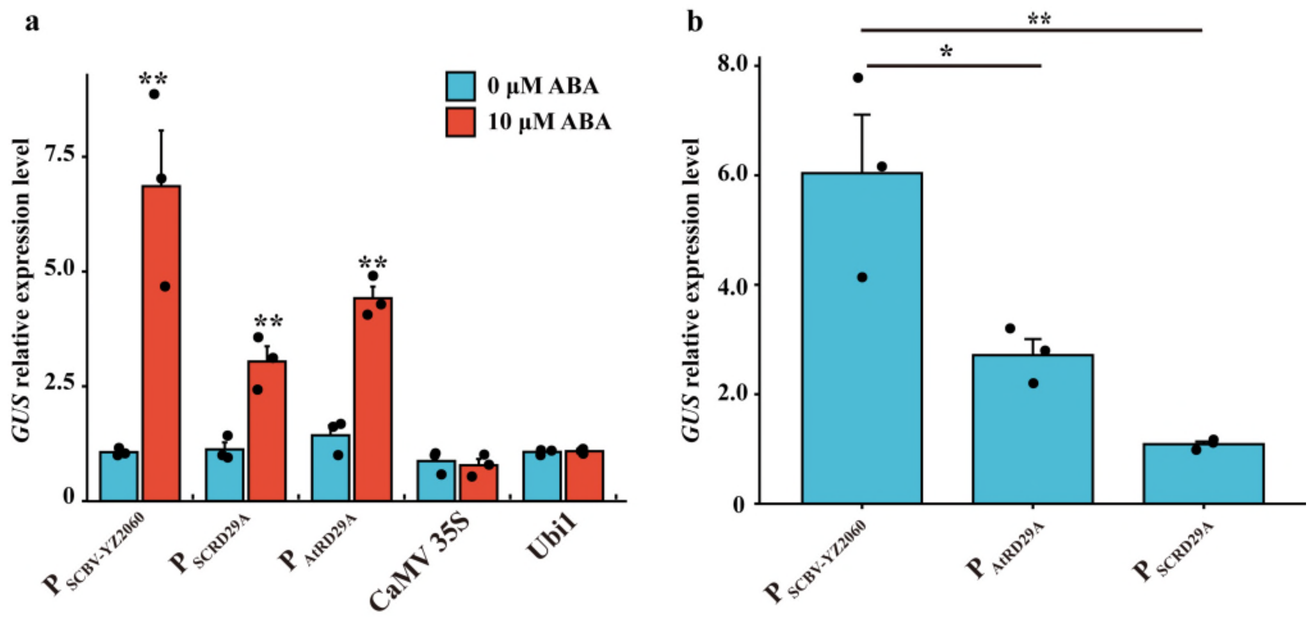

Supplementary Figure 3. Transcriptional expression of the *GUS* gene driven by five different promoters ($P_{SCBV\text{-}YZ2060}$, $P_{ScRD29A}$, $P_{AtRD29A}$, CaMV 35S, and Ubi1) in wild-type *Arabidopsis* protoplasts based on a RT-qPCR assay. (a) Comparison of expression levels of the *GUS* gene driven by five different promoters after treatment of protoplasts with 0 or 10 μM ABA. Values are the means (± standard errors) of three biological replicates at 8 h post treatment. For each promoter, values that are significantly different at $P = 0.01$ (Student's T-test) are indicated by two asterisks. (b) Comparison of expression levels of the *GUS* gene driven by three different promoters ($P_{SCBV\text{-}YZ2060}$, $P_{ScRD29A}$, $P_{AtRD29A}$) after treatment of protoplasts with 10 μM ABA. Relative gene expression of each promoter was determined versus their respective 0 μM ABA control. Values are the means (± standard errors) of three biological replicates at 8 h post treatment. Values that are significantly different at $P = 0.05$ and $P = 0.01$ (Student's T-test) are indicated by one and two asterisks, respectively.

```
1      AAGAACCAGT ACTAATGTGT GCATGCAGGA AACCGGCGGT TCTCTTCACC TCAGGAACAA
                                                                 TSS1
61     GGAACAATCC AAGCCGGAAG TTCTACAAAT GTGTGGCAAA TCAATGCCAT TGCTGGTACT
121    GGAAGGATCT CATTGAAGCT TACGTCCAAG ATCGCATTGA AGAGTTCATG GTCGACAACT
                TATA box
181    TCGACAGTAA AATGAACATA TCAGAAGCTT CAACAAGTCA AGCCAAGCCG GAGATTGAAG
241    AAGATCCGTT AGAAAATCTT CGATCAAGCG TCATTGATAG GCCAAGGCCT AGCGATGAAC
301    ATTTCAAGCC AGGGTATGAA TATCCTCAGT GTCCGGAGTA CGTTCAAGAA GAACTCGCCA
361    ATAGGTTAAT GACCTATGAA GAATACCTCA AGATGATTCA AAGTGAAGAG CACCTCCATC
421    AGCAAAACTC TTTGCAGAAG ATTGCTGAAG ATTATCCAAG CCCACCATGG GGAGAACTGG
481    ACCTCTATTG CCATGAAGAC CCAGACTTGG TGTACGAAGA CGCCCGCACA GAAGATCTGC
541    TCGACCTTGA AGACGTCATC GATGACATCA GAAGCTGAAG AAACGTCACT GCTGACCTCA
601    AGACGCATCA AGCGGAGCGT GAAGGACCCA TTCAGTGGAC CTCACCACTG AAGAAGAATC
661    TCAACTTTCG GCGCAATAAT GCGTTAGGTG TGCCCGGCAC CATGTTCGGT GCGATGTATC
                  ABRE-2
721    GAGTCTGTCG GTTGTACGTG TCCAGCACCT TTGTTCGGTG CGTGTCCTTT TCGGGCATCT
                             ABRE-1
781    GTGCCCATCT TCCTTTGTCG GCCACGTTGC CTTTGCTTAG CCCTTACGCG AAGCATAGCG
                                    TATA box                 TSS2
841    CTCGGCCCTG GTGTGCCCTC TGCCTATATA AGGCATGGAT GTAAGGCTCT TACACTCATC
901    GGTAGTTCAC CACATGAGTA TTTGAGCTTG TTTC
```

Supplementary Figure 4. Sequence analysis of promoter $P_{SCBV\text{-}YZ2060}$. The two putative transcriptional start sites, TSS1 and TSS2, are marked in red and blue bold letters, respectively. The two TATA-boxes (TATAAAT and ATATAA) are boxed in red rectangles. The two putative ABRE *cis*-acting regulatory elements (ABRE-2 and ABRE-1) are underlined in orange and green, respectively.

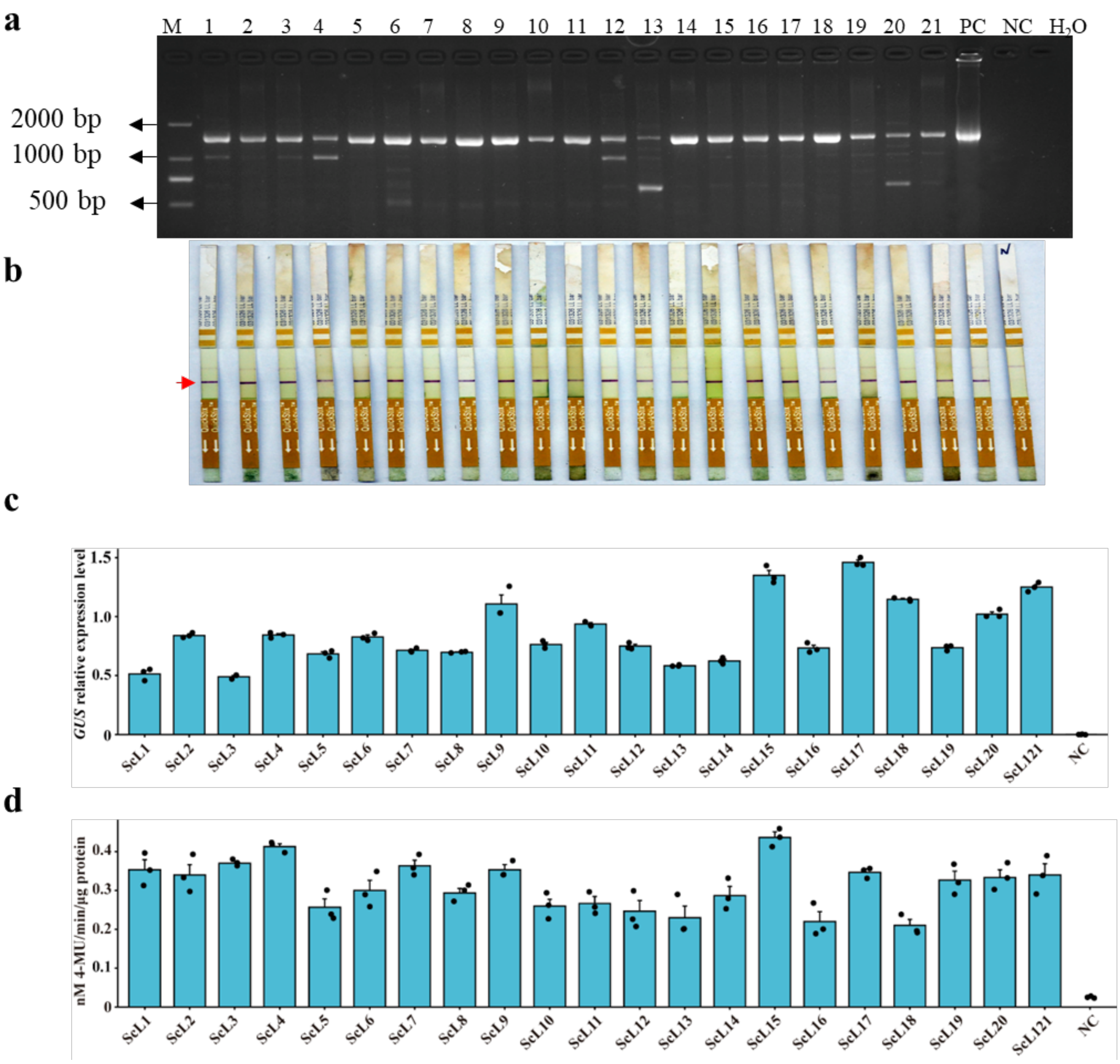

Supplementary Figure 5. Identification of $P_{SCBV\text{-}YZ2060}$:GUS transgenic sugarcane lines. (a) PCR identification of transformed sugarcane. Lanes 1-21, transgenic sugarcane lines; M = DL2000 DNA Marker; PC = positive control ($P_{SCBV\text{-}YZ2060}$:GUS plasmid); NC = negative control (wild-type sugarcane, cultivar ROC22). A positive amplification resulted in production of a band of about 1805 bp. (b) Identification of transformed sugarcane with the PAT/bar strip test; *bar*-positive test lines are indicated by a red arrow. The first 21 strips from the left are transformed sugarcane lines; strip 22 = positive control and strip 23 = negative control. (c) and (d) RT-qPCR of *GUS* gene and GUS fluorometric assay, respectively. The first 21 columns from the left are transformed sugarcane lines. NC = negative control. Each column represents the mean value (± standard error) of three technological replicates.

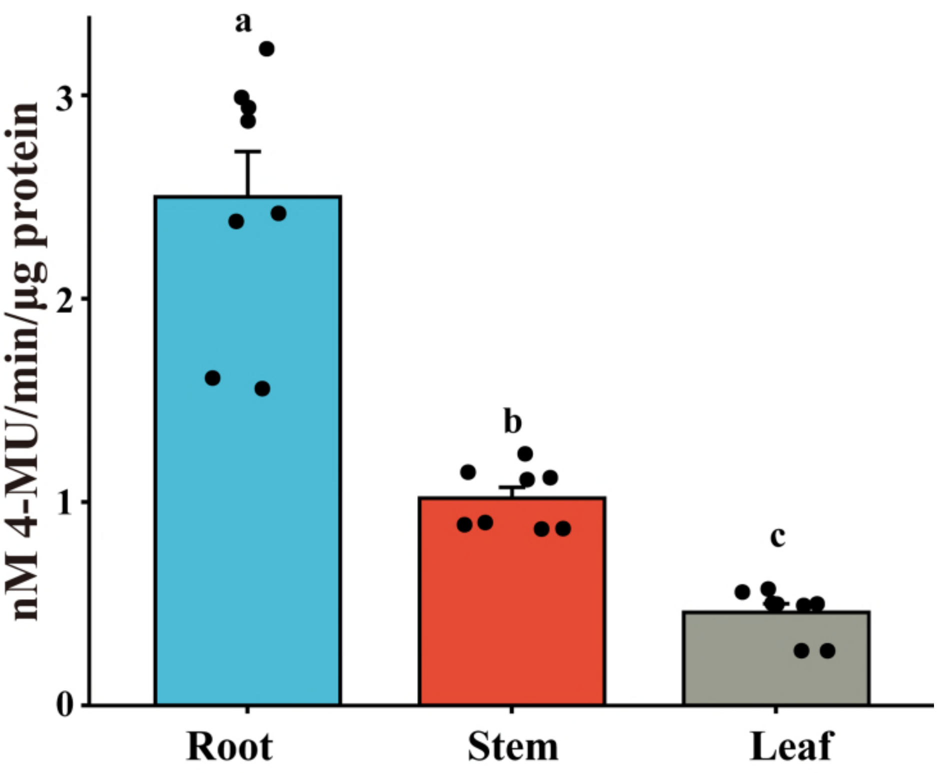

Supplementary Figure 6. GUS protein activity in root, stem, and leaf tissues of sugarcane transformed with $P_{SCBV-YZ2060}$:GUS. Values are the means (± standard errors) of four sugarcane transgenic lines and three technological replicates. Mean values with the same letter are not significantly different at *P* = 0.05 according to Duncan's multiple range test.

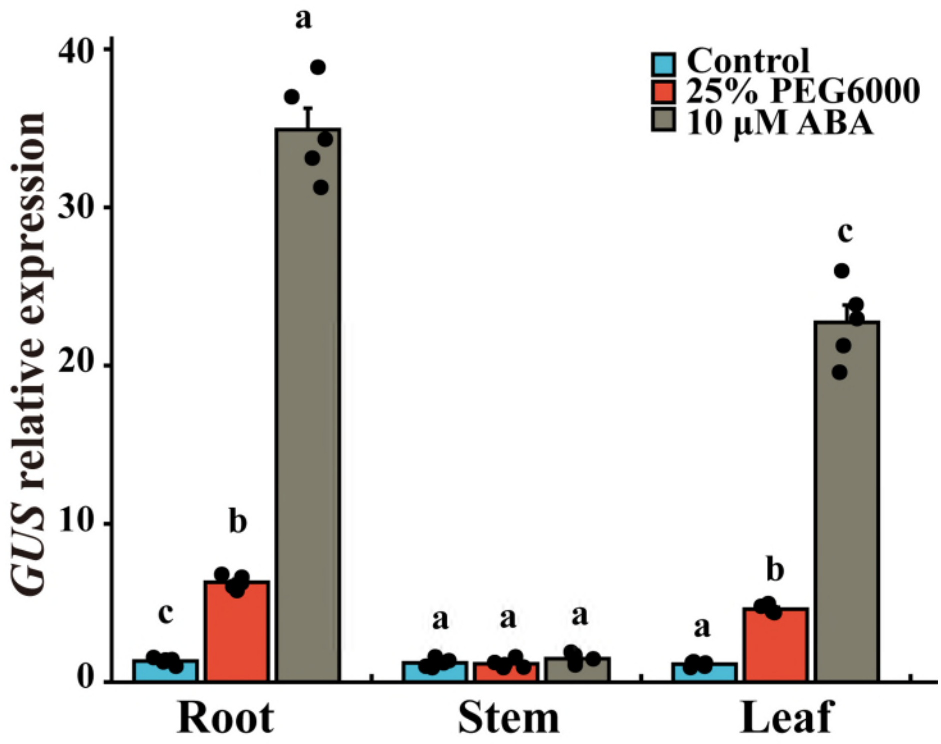

Supplementary Figure 7. Expression of gene *ScbZIP72* in roots, stem, and leaf tissues of sugarcane transformed with promoter vector $P_{SCBV\text{-}YZ2060}$:GUS and subjected to 25% PEG6000 and 10 μM ABA treatments. Control = no treatment. Values are the means (± standard errors) of six transgenic lines and three technological replicates. For each tissue, mean values with the same letter are not significantly different at *P* = 0.05 according to Duncan's multiple range test.

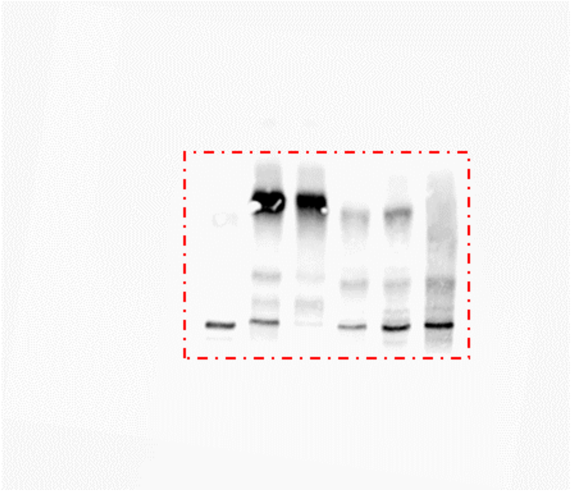

Supplementary Figure 8. Uncropped EMSA image showing that transcription factor ScbZIP72 targets directly ABRE motif sL by binding to this motif. This EMSA image was obtained as described in the materials and methods section. The dotted rectangle indicates the part of the image used in the corresponding panel of Fig. 7c.

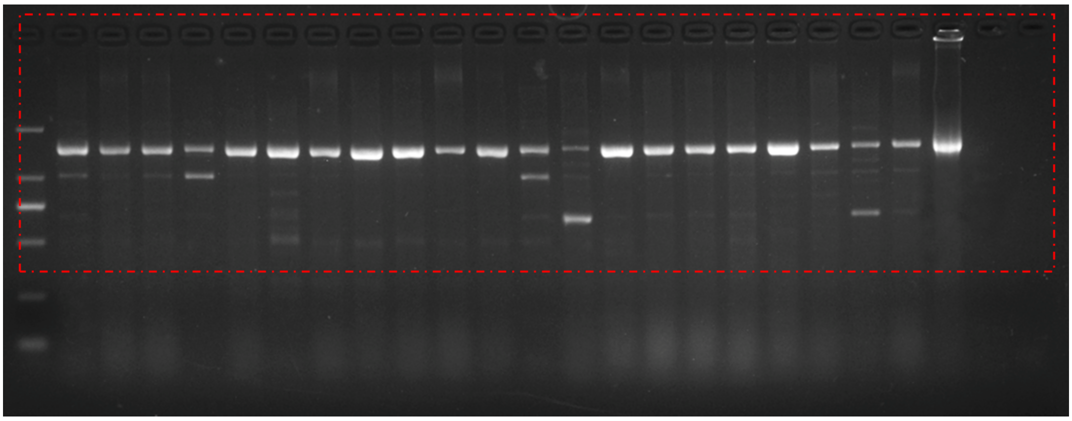

Supplementary Figure 9. Uncropped gel image showing that positive transformed sugarcane are identified by PCR. This gel image was obtained as described in the materials and methods section. The dotted rectangle indicates the part of the image used in the corresponding panel of Supplementary Fig. 5a.

References

Janiga, P. K., Nithya, K., & Viswanathan, R. Dynamics of genetic diversity among Indian sugarcane bacilliform virus species and implications of associated recombination events in the virus, *Sugar Tech* **25**, 705–716 (2023).
