# Supplementary material for: Identification of a sugarcane bacilliform virus promoter that is activated by drought stress in plants
Source: Commun Biol. 2024 Mar 26;7:368. doi: 10.1038/s42003-024-06075-y (PMC10965894; doi:10.1038/s42003-024-06075-y)
Supplement: Supplementary file 3 — Description of Additional Supplementary Files [file 42003_2024_6075_MOESM3_ESM.pdf]

Description of Additional Supplementary Files

File name: Supplementary data 1

Description: List of primers used for plasmid constructions in this study.

File name: Supplementary data 2

Description: Source data for graph from Main manuscript file.

File name: Supplementary data 3

Description: Source data for graph from Supplementary Information.
